# Dynamical Analysis of an SEIT Epidemic Model with Application to Ebola Virus Transmission in Guinea

**DOI:** 10.1155/2015/582625

**Published:** 2015-07-02

**Authors:** Zhiming Li, Zhidong Teng, Xiaomei Feng, Yingke Li, Huiguo Zhang

**Affiliations:** ^1^College of Mathematics and System Sciences, Xinjiang University, Urumqi 834800, China; ^2^Department of Mathematics, Yuncheng University, Yuncheng 044000, China; ^3^College of Mathematics and Physics, Xinjiang Agricultural University, Urumqi 830052, China

## Abstract

In order to investigate the transmission mechanism of the infectious individual with Ebola virus, we establish an SEIT (susceptible, exposed in the latent period, infectious, and treated/recovery) epidemic model. The basic reproduction number is defined. The mathematical analysis on the existence and stability of the disease-free equilibrium and endemic equilibrium is given. As the applications of the model, we use the recognized infectious and death cases in Guinea to estimate parameters of the model by the least square method. With suitable parameter values, we obtain the estimated value of the basic reproduction number and analyze the sensitivity and uncertainty property by partial rank correlation coefficients.

## 1. Introduction

Ebola virus disease (EVD) is first identified in the Democratic Republic of Congo (formerly Zäire) in 1976. It is a lethal viral hemorrhagic fever and can cause a high case fatality rate lying between 50 and 90% [1, 2]. Since 1976, the outbreak of Ebola epidemic has happened more than 20 times, most of which appeared in Africa. Sudan (1976, 1979, and 2004), Democratic Republic of Congo (1976, 1977, 1995, 2007, 2008, and 2012), Gabon (1994, 1996, 2001, and 2002), Republic of Côte d'Ivoire (1994), Uganda (2000, 2007, 2011, and 2012), and Republic of the Congo (2001, 2002, 2003, and 2005) have reported EVD epidemics [3, 4].

Mathematical modeling has emerged as an important tool for gaining understanding of the dynamics of the spread of EVD. Reference [5] established a stochastic discrete-time susceptible-exposed-infectious-recovered (SEIR) model to estimate parameters from daily incidence and mortality time series for an outbreak of Ebola in the Democratic Republic of Congo in 1995. Different from [5], reference [1] used both onset and death date to constrain the optimization of SEIR model parameters by Bayesian inference. To develop a better understanding of Ebola transmission dynamics, [6] introduced a compartmental model to quantify transmission in different settings (illness in the community, hospitalization, and traditional burial). Further, [7] used the above model to analyze the temporal dynamics of Ebola.

The current Ebola outbreak began in December 2013 in Guinea [8], initially in the Prefecture of Gueckedou, and shortly spread to other West African countries such as Liberia, Sierra Leone, Nigeria, and Senegal [6, 9–11]. The outbreak is the largest to date: as of 25th January 2015, 22,092 cases have been reported by World Health Organisation (WHO), as well as 8,810 deaths [12], which contain 2,917 infected cases and 1,910 deaths of Guinea. Reference [13] analyzed transmission dynamics of EVD in Nigeria and showed the time window for successful containment of EVD outbreaks caused by infected air travelers. Reference [14] designed a model that is formulated by splitting the total population into two main subgroups, namely a subgroup of individuals in the community and another for those in health-care settings.

To assess the effect these various intervention strategies could have on controlling the spread of Ebola virus, we develop a mathematical model for transmission, fitted probabilistically to epidemiological data of reported cases in Guinea. Experimental vaccines and treatments for Ebola are under development, but they have not yet been fully tested for safety or effectiveness. Thus, we establish an SEIT model and apply it to analyze the spread of EVD in Guinea, where *S*, *E*, *I*, and *T* denote the number of the susceptible, exposed in the latent period, infectious, and treated/recovered population, respectively. For the SEIT model, [15–17] constructed a discrete version with latent age structure to investigate tuberculosis transmission. Along the paper, we assume that infected individuals in SEIT model can develop EVD by contacting the exposed in the latent period or infectious individuals.

This paper is organized as follows.  Section 2 introduces an SEIT model by a four-dimensional differential equations system. In Section 3, the basic reproduction number *R*_0_ is defined. Based on *R*_0_ < 1 and *R*_0_ > 1, the local stability of the disease-free equilibrium and endemic equilibrium is obtained according to Hurwitz criterion. In Section 4, by the least square method we propose the estimation of parameters by onset and death data in Guinea, which can be used to calculate *R*_0_. Moreover, we provide the sensitivity and uncertainty analysis of *R*_0_.  Section 5 presents our concluding remark.

## 2. Model Formulation

The transmission of EVD is by direct contact with body fluids, secretions, tissues, or semen from the infectious individuals [1], which starts with acute fever, diarrhea that can be bloody, and vomiting followed by headache, nausea, and abdominal pain [2]. Its incubation period ranges from 2 to 21 days (5–12 days in most cases) [1]. Since the diseases had caused the loss of thousands of lives and brought great pain to families, there have been lots of mathematical models for gaining understanding of the dynamics of the spread of EVD [1, 2, 6, 7, 18, 19]. In this work, we establish an SEIT model to extend the SEIR and SEIRS types and apply the model to describe the dynamics of EVD during 2014 outbreak in Guinea.

**Figure 1:**
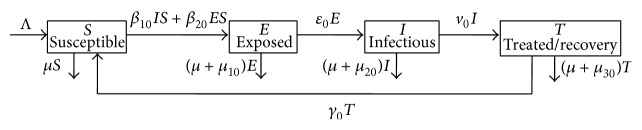
Transfer diagram of SEIT epidemic model.

The total host population is partitioned into susceptibles, exposed (in the latent period), infectious, and treated/recovered individuals, respectively, denoted by *S*(*t*), *E*(*t*), *I*(*t*), and *T*(*t*) at time *t*. After one unit time, a susceptible individual can be infected through contacting with the exposed or infectious individuals and enter the latent class or is still in the susceptible class or dies. A latent individual may become infectious and enter the infectious class or still stay in the latent class or die. An infectious individual may be treated and enter the treated/recovery class or stay in the infectious class or die. For a treated individual, he (or she) may recover by effective treatment. The recovered individual from Ebola depends on good supportive care and the patients immune response. People who recover from Ebola infection develop antibodies that last for at least 10 years, possibly longer. However, it is not known whether people who recover are immune for life or whether they can become infected with a different species of Ebola [20]. Thus, we consider that the recovered individual may enter the susceptible class. Otherwise, the treated individual may stay in the treated/recovered class, or die.  Figure 1 shows the relationship between the four variables of our SEIT model.

Using Figure 1, we formulate the following SEIT model, which is a four-dimensional differential equations model:

(1) S˙=Λ−β10IS−β20ES−μS+γ0T,E˙=β10IS+β20ES−ε0E−μ+μ10E,I˙=ε0E−v0I−μ+μ20I,T˙=v0I−γ0T−μ+μ30T,

where Λ is the recruitment rate; the positive parameter *μ* is the rate of natural death; *μ*_10_, *μ*_20_, and *μ*_30_ are nonnegative constants and denote rates of disease-caused death. Parameters *β*_10_ and *β*_20_ are the rate of the efficient contact in the infected, latent, and exposed period; *ε*_0_ and *v*_0_, respectively, denote the transfer rates between the exposed and the infectious, the infectious and treated infectious; and *γ*_0_ denotes the rate of the effectively treated individuals. In model (1), when *γ*_0_ = 0, the SEIT model is an SEIR type. If *γ*_0_ ≠ 0, the SEIT model is an SEIRS type. Thus, the SEIT model is a general version of SEIR or SEIRS type.

For convenience, denote *β*_1_ = *β*_10_/*μ*, *β*_2_ = *β*_20_/*μ*, *μ*_1_ = *μ*_10_/*μ*, *μ*_2_ = *μ*_20_/*μ*, *μ*_3_ = *μ*_30_/*μ*, *ε* = *ε*_0_/*μ*, *v* = *v*_0_/*μ*, and *γ* = *γ*_0_/*μ*. Further, define

(2) ω1=1+ɛ+μ1,ω2=1+v+μ2,ω3=1+γ+μ3.

Let *τ* = *μt*; then model (1) is equivalent to the following form:

(3) dSdτ=Λμ−β1IS−β2ES−S+γT,dEdτ=β1IS+β2ES−ω1E,dIdτ=ɛE−ω2I,dTdτ=vI−ω3T.

## 3. Equilibria and Local Stability

It is clear that model (3) always has a disease-free equilibrium *P*_0_(*S*_0_, 0,0, 0) with *S*_0_ = Λ/*μ*. Let *P* ^*∗*^ = (*S* ^*∗*^, *E* ^*∗*^, *I* ^*∗*^, *T* ^*∗*^) be the endemic equilibrium; then we have

(4) Λμ−β1I∗S∗−β2E∗S∗−S∗+γT∗=0,β1I∗S∗+β2E∗S∗−ω1E∗=0,ɛE∗−ω2I∗=0,vI∗−ω3T∗=0.

Define the basic reproduction number as

(5) $$\begin{array}{c} R_{0}=\frac{\Lambda \left(ɛ\beta_{1}+\omega_{2}\beta_{2}\right)}{\mu \omega_{1}\omega_{2}}. \end{array}$$

Based on (2), we know that *ω*_1_, *ω*_2_, *ω*_3_ > 0 and *ω*_1_ *ω*_2_ *ω*_3_ − *ɛγv* > 0. When *R*_0_ > 1, solving (4) we obtain the endemic equilibrium *P* ^*∗*^:

(6) S∗=ω1ω2ɛβ1+ω2β2,E∗=ω2ω3Λɛβ1+ω2β2−μω1ω2μω1ω2ω3−ɛγvɛβ1+ω2β2,I∗=ɛω3Λɛβ1+ω2β2−μω1ω2μω1ω2ω3−ɛγvɛβ1+ω2β2,T∗=ɛvΛɛβ1+ω2β2−μω1ω2μω1ω2ω3−ɛγvɛβ1+ω2β2.

Now, we discuss the local stability of equilibria. Firstly, on the stability of disease-free equilibrium *P*_0_ we have the following result.

**Theorem 1 .**

If *R*_0_ < 1, then disease-free equilibrium *P*_0_ = (Λ/*μ*, 0,0, 0) is locally asymptotically stable.

**Proof**

The Jacobian of model (3) is

(7) $$\begin{array}{c} J=\left(\begin{array}{cccc} -\beta_{1}I-\beta_{2}E-1 & -\beta_{2}S & -\beta_{1}S & \gamma \\ \beta_{1}I+\beta_{2}E & \beta_{2}S-\omega_{1} & \beta_{1}S & 0\\ 0 & ɛ & -\omega_{2} & 0\\ 0 & 0 & v & -\omega_{3} \end{array}\right). \end{array}$$

Thus, the Jacobian at point *P*_0_ is

(8) $$\begin{array}{c} J\left(\;P_{0}\;\right)=\left(\begin{array}{cccc} -1 & -\frac{\beta_{2}\Lambda }{\mu } & -\frac{\beta_{1}\Lambda }{\mu } & \gamma \\ 0 & \frac{\beta_{2}\Lambda }{\mu }-\omega_{1} & \frac{\beta_{1}\Lambda }{\mu } & 0\\ 0 & ɛ & -\omega_{2} & 0\\ 0 & 0 & v & -\omega_{3} \end{array}\right) \end{array}$$

and its characteristic equation is given by det⁡(*λI* − *J*(*P*_0_)) = 0, where *I* is the unit matrix, since

(9) det⁡λI−JP0=λ+1β2Λμβ1Λμ−γ0λ−β2Λμ+ω1−β1Λμ00−ɛλ+ω2000−vλ+ω3=λ+1·λ+ω3λ−β2Λμ+ω1λ+ω2−ɛβ1Λμ.

Clearly, there exist two roots *λ*_1_ = −1 and *λ*_2_ = −*ω*_3_, and other roots satisfy

(10) f1λλ−β2Λμ+ω1λ+ω2−ɛβ1Λμ=λ2+a1λ+a2,

where

(11) a1=ω1+ω2−β2Λμ,a2=ω1ω2−Λμβ2ω2+ɛβ1.

If *R*_0_ = Λ(*ɛβ*_1_ + *ω*_2_ *β*_2_)/*μω*_1_ *ω*_2_ < 1, then *ω*_1_ > Λ*ɛβ*_1_/*μω*_2_ + Λ*β*_2_/*μ* and *ω*_1_ *ω*_2_ > Λ*ɛβ*_1_/*μ* + (Λ*β*_2_/*μ*)*ω*_2_. Clearly, *a*_1_, *a*_2_ > 0 and *a*_1_ *a*_2_ > 0. According to* Hurwitz criterion*, all roots of *f*_1_(*λ*) have negative real parts. Hence, disease-free equilibrium *P*_0_ is local asymptotical stability.

Next, on the stability of endemic equilibrium *P* ^*∗*^, we have the result as follows.

**Theorem 2 .**

If *R*_0_ > 1, then endemic equilibrium *P* ^*∗*^ is locally asymptotically stable.

**Proof**

By (7), the matrix of the linearization of model (3) at equilibrium *P* ^*∗*^ is

(12) JP∗=−β1I∗−β2E∗−1−β2S∗−β1S∗γβ1I∗+β2E∗β2S∗−ω1β1S∗00ɛ−ω2000v−ω3.

The corresponding characteristic equation is

(13) f2λdet⁡λI−JP∗=λ4+b1λ3+b2λ2+b3λ+b4,

where

(14) b1=β1I∗+β2E∗−β2S∗+ω1+ω2+ω3+1,b2=ω1+ω2+ω3β1I∗+β2E∗+1−1+ω2+ω3β2+ɛβ1S∗+ω1ω2+ω3+ω2ω3,b3=ω2ω3+ω1ω2+ω3β1I∗+β2E∗−ω2ω3+ω2+ω3β2+ɛ1+ω3β1S∗+ω1ω2ω3+ω2+ω3+ω2ω3,b4=ω1ω2ω3−ɛγvβ1I∗+β2E∗.

Denote *A* = (Λ(*ɛω*_3_ *β*_1_ + *ω*_2_ *ω*_3_ *β*_2_) − *μω*_1_ *ω*_2_ *ω*_3_)/*μ*(*ω*_1_ *ω*_2_ *ω*_3_ − *ɛγv*). Based on (6), we have

(15) $$\begin{array}{c} \beta_{1}I^{*}+\beta_{2}E^{*}=\frac{ɛ\beta_{1}+\omega_{2}\beta_{2}}{ɛ}I^{*}=A. \end{array}$$

Thus,

(16) b1=A+ɛω1β1ɛβ1+ω2β2+ω2+ω3+1,b2=ω1+ω2+ω3A+ɛω1β1+ɛω1ω3β1ɛβ1+ω2β2+ω2+ω3+ω2ω3,b3=ω2ω3+ω1ω2+ω3A+ω2ω3+ɛω1ω3β1ɛβ1+ω2β2,b4=ω1ω2ω3−ɛγvA.

The calculation of *b*_*i*_, *i* = 1,2, 3,4, is placed in Appendix A.

In order to obtain the local asymptotical stability of *P* ^*∗*^, we need to verify the following conditions according to Hurwitz criterion: (i) *b*_*i*_ > 0 for *i* = 1,2, 3,4; (ii) *b*_1_ *b*_2_ − *b*_3_ > 0; and (iii) *b*_3_(*b*_1_ *b*_2_ − *b*_3_) − *b*_1_^2^ *b*_4_ > 0:

(i) Since *β*_1_, *β*_2_, *ω*_1_, *ω*_2_, *ω*_3_ > 0 and *ω*_1_ *ω*_2_ *ω*_3_ − *ɛγv* > 0, it is easy to get *b*_1_, *b*_2_, *b*_3_, *b*_4_ > 0.
(ii) For *b*_1_ *b*_2_ − *b*_3_, we have
  (17) b1b2−b3=ω1+ω2+ω3A2+ɛω1β1+ɛω1ω3β1ɛβ1+ω2β2A+ω1+ω2+ω3A+ɛω1β1ɛβ1+ω2β2ω1+ω2+ω3A+ω2+ω3ω2+ω3+1A+ɛω1β1ɛβ1+ω2β2ω2+ω3+ω2ω3+ɛω1β1ɛω1β1+ɛω1ω3β1ɛβ1+ω2β22+ɛω1ω2β1+ɛω1ω2ω3β1ɛβ1+ω2β2+ɛω1ω32β1ɛβ1+ω2β2+ɛω1β1+ɛω1ω3β1ɛβ1+ω2β2+ω2+ω3ω2+ω3+1+ω2ω3ω2+ω3.
 Clearly, *b*_1_ *b*_2_ − *b*_3_ > 0.
(iii) Based on (16) and (ii), we have *b*_3_(*b*_1_ *b*_2_ − *b*_3_) − *b*_1_^2^ *b*_4_ > 0. Because the expression of *b*_3_(*b*_1_ *b*_2_ − *b*_3_) − *b*_1_^2^ *b*_4_ is too long, we place it in Appendix B. According to* Hurwitz criterion*, all roots of *f*_2_(*λ*) have negative real parts. Hence, endemic equilibrium *P* ^*∗*^ is local asymptotical stability.

## 4. Application of the Model

In this section, model (1) will be applied to analyze the characteristics of EVD, which is equivalent to system (3). In order to estimate its parameters and calculate the basic reproduced number *R*_0_, we use the onset and death data of EVD to fit the observed variables by least square method.

### 4.1. Data Sets

The 2014 Ebola outbreak began in Guinea. The patient zero is a 2-year-old toddler named Emile Ouamouno, who lived in the village of Meliandou, sitting close to Guinea's borders with Sierra Leone and Liberia. The boy was infected in December 2013 and it is not clear exactly how he got infected [8]. In December, Emile had a fever, black stool, and started vomiting. Four days later, on December 6, he was dead. Within a month, so were his young sister, his mother, and his grandmother.

**Figure 2:**
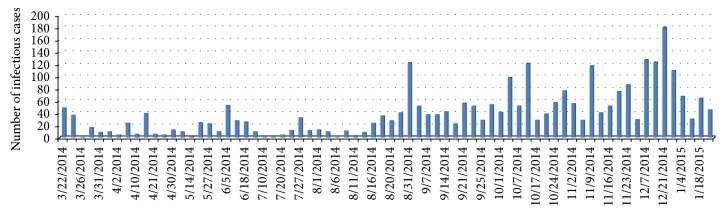
Number of recognized infectious cases.

**Figure 3:**
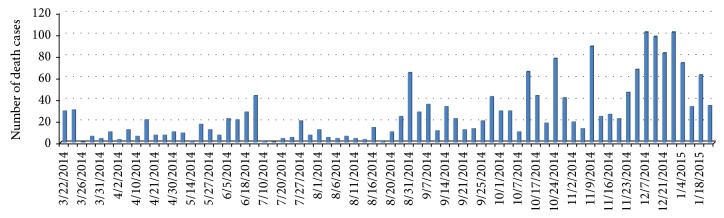
Number of recognized death cases.

In March 22, 2014, the Ministry of health of Guinea has reported the acute infectious disease named EVD, which began with fever, severe diarrhea, vomiting, and high case fatality rate (59%). WHO publicly announced outbreak of EVD on its web site on March 23; 49 cases and 29 deaths were officially reported in March 22 [21]. By January 25, 2015, 2,917 reported cases of Ebola in Guinea were identified, of which 1,910 individuals had died. From the first case in December 1, 2013, to January 18, 2015, in Guinea, the Ebola epidemics lasted 400 days, but the recorded data starting time and evolution of the epidemic is March 22, 2014. Therefore, the onset and death data were collected from March 22, 2014, to January 25, 2015 [22]; see Figures 2 and 3.

### 4.2. Least Square Method for Parameters Estimation

In this subsection, we use the least square method to estimate the parameters of model (1). The spread of EVD started in December 2013, in which the whole population of Guinea was 11,745,000 in this year [23]. Based on the data sets (see Figures 2 and 3), we know that *I*(0) = 49, *T*(0) = 49 − 29 = 20, and *S*(0) = 11,745,000 − 49 = 11,744,951. Moreover, since Ebola incubation period ranges from 5 to 12 days in most cases [1], then we take 5 days as the incubation period. Therefore, *E*(0) = 37 (that is the reported infected cases at the fifth day minus the reported infected cases at the first day).

**Figure 4:**
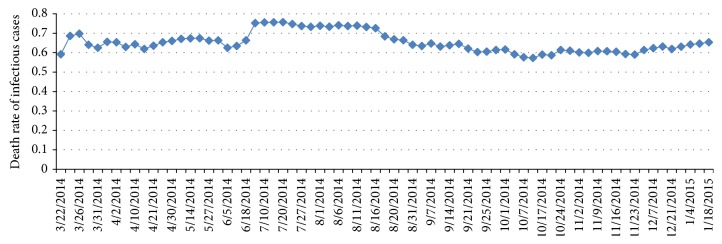
Ebola disease-caused death rate of recognized infectious cases.

In model (1), there are ten parameters that correspond to the recruitment rate Λ, the natural and disease-caused death rates *μ*, *μ*_10_, *μ*_20_, and *μ*_30_, the efficient contact rates in the infected and the latent period *β*_10_ and *β*_20_, and the transfer rates *ε*_0_, *v*_0_, and *γ*_0_. Among these parameters, parts of them need to be fixed and others will be estimated as follows:

(P1)
The recruitment rate Λ can be given by birth rate, which is the total number of births per 1,000 of a population in a year. Based on [24–26], the 2014 birth rate of Guinea is 0.03602. Thus, Λ = (0.03602/365) × 11,744,951 = 1,159.
(P2)
The natural death rate *μ* is typically expressed in units of deaths per 1,000 individuals per year. Thus, we use the natural death rate *μ* = 0.0097/365 = 2.657 × 10^−5^ of Guinea in 2013 [27]. For two disease-caused death rates *μ*_10_ in the latent period and *μ*_30_ in treated/recovery period, their values are smaller than that of *μ*_20_ in the infectious period. Thus, we assume that *μ*_10_ = *μ*_30_ = 0. The rate *μ*_20_ will be estimated. In order to provide the value range of *μ*_20_, we use the method in which the accumulative death number is divided by the accumulative cases. Then, it is obtained that 0.5 ≤ *μ*_20_ ≤ 0.8 (see Figure 4).
(P3)
In this paper, we consider that Ebola virus during the latent and exposed period is contagious. In general, the efficient contact rates *β*_10_ and *β*_20_ are different. In order to reduce the risk of human-to-human transmission, WHO raises the awareness of the risk factors of Ebola infection and the protective measures [28, 29]. Thus, we consider that 0 < *β*_10_ ≤ *β*_20_ < 1 and all of them will be estimated.
(P4)
Since Ebola incubation period ranges from 2 to 21 days (5–12 days in most cases) [1], the transfer rate *ε*_0_ is set following the constraint 2 < 1/*ε*_0_ < 21; that is, 0.04761 < *ε*_0_ < 0.5. For the transfer rates *v*_0_, we will estimate it and let it satisfy 0 < *v*_0_ < 1.
(P5)
Based on [2, 5], we know that the effective treated rate 3.5 < 1/*γ*_0_ < 10.7; that is, 0.093 < *γ*_0_ < 0.2857.

Based on the conditions (P1)–(P5), parameters of model (1) to be estimated are *β*_10_, *β*_20_, *μ*_20_, *ε*_0_, *v*_0_, and *γ*_0_. We list their estimated values in Table 1.

### 4.3. Numerical Simulations

**Figure 5:**
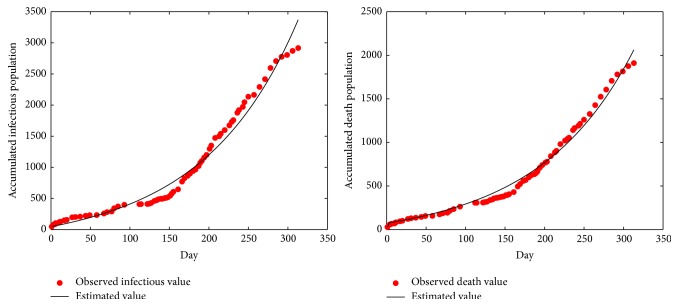
Real values and fitting values of the accumulative infectious cases and death cases.

Using the parameter values in Table 1, numerical simulations of model (1), respectively, give the comparison curves between the real values and fitting values of the accumulative infectious cases and accumulative death cases from March 22, 2014, to January 25, 2015 [22]; see Figure 5.

Through Figure 5, we observed that the EVD infection still does not get the effective control. This point can be confirmed by threshold value *R*_0_. Based on the estimated values of parameters in Table 1, we have *R*_0_ = Λ(*ɛβ*_1_ + *ω*_2_ *β*_2_)/*μω*_1_ *ω*_2_ = 4.16, where *β*_*i*_ = *β*_*i*0_/*μ*, *i* = 1,2. Since *R*_0_ > 1, by Theorem 2, endemic equilibrium *P* ^*∗*^ of model (1) is locally asymptotically stable. This indicates that EVD infection still infects humans and will be endemic in Guinea without the effective control measures.

### 4.4. Sensitivity and Uncertainty Analysis of *R*_0_

Because *R*_0_ is an important threshed value for the spread of EVD, we perform uncertainty and sensitivity analysis of *R*_0_ in model (1) using partial rank correlation coefficients (PRCCs) [30]. Based on (1), (2), and (5), we get the expression of *R*_0_ as follows:

(18) $$\begin{array}{c} R_{0}=\frac{\Lambda \left(\varepsilon_{0}\beta_{10}+\beta_{20}\left(\mu +v_{0}+\mu_{20}\right)\right)}{\mu \left(\mu +v_{0}+\mu_{20}\right)\left(\mu +\varepsilon_{0}+\mu_{10}\right)}. \end{array}$$

**Figure 6:**
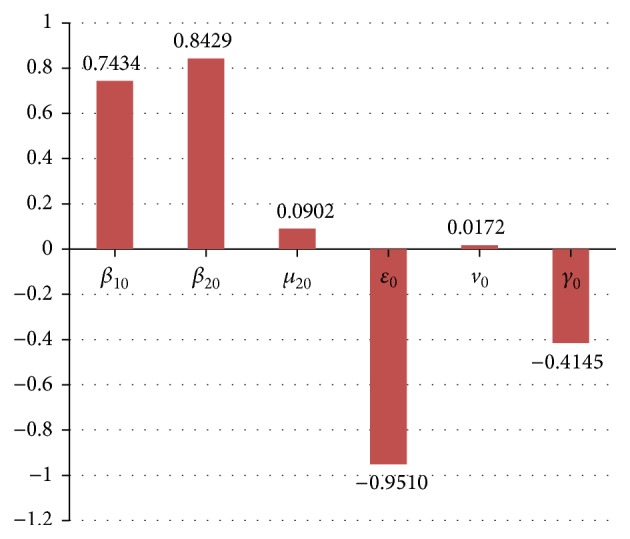
PRCCs for the effect of six parameters on *R*_0_.

Among all parameters of model (1), we only analyze the influence of seven parameters in determining the magnitude of *R*_0_ except the fixed numbers Λ, *μ*, *μ*_10_, and *μ*_30_, which have been discussed in Section 4.2. The ordering of these PRCCs directly corresponds to the level of statistical influence, the impact that uncertainty in the estimate of a parameter has on the variability of *R*_0_. A positive PRCC value indicates that an increase in that parameter leads to an increase in *R*_0_, while a negative value shows that increasing that parameter decreases *R*_0_. For the Ebola SEIT model, seven parameters were significantly different from 0 (*p* value < 0.05). Among these parameters, *β*_10_, *β*_20_, *μ*_20_, and *v*_0_ have a positive influence on *R*_0_, while *ε*_0_ and *γ*_0_ have a negative influence on *R*_0_; see Figure 6.

## 5. Conclusions

In this paper, we establish the SEIT model to analyze the dynamical properties of EVD transmission in Guinea. One of the reasons is that there is still no effective treatments for EVD and the current response is only support treatment [31]. In particular, if parameters *γ*_0_ = 0 or *γ*_0_ ≠ 0, the SEIT model will become the SEIR or SEIRS types.

The SEIT model is formed by four-dimensional differential equations. In order to understand the transmission of Ebola virus, we discuss the local stability of the disease-free equilibrium and the endemic equilibrium by basic reproduction number *R*_0_ < 1 and *R*_0_ > 1. Since there exist ten parameters in the expression of *R*_0_, it is important to estimate the value of each parameter. By the least square method and the recorded data in Guinea we obtain the estimation of seven parameters except for four fixed constants in Table 1. In Table 1, we observe that the effective treated rate is only 0.2999, and the disease-caused death rate in infectious period is 0.6647. Thus, it is urgent to provide the effective vaccines to cure the infectious people and protect the susceptible.

With suitable parameter values, we obtain estimation value of *R*_0_. The result shows that the Ebola virus still infects people in Guinea and does not disappear in short time. Without the effective control measures, the EVD may be endemic in Guinea. By the PRCCs method, we analyze the sensitivity and uncertainty of *R*_0_. The result shows that the rates of the efficient contact, especially in latent and exposed period, lead to the significant increase in *R*_0_ (see Figure 6).

On the other hand, it would be interesting to study more properties of the present model. In particular, a study involving both stability properties of pulse vaccination strategy and global stability is worth pursuing. We leave these for future consideration.

## Supplementary Material

This paper establishes an SEIT epidemic model (susceptible, exposed in the latent period, infectious, treated/recovery) to analyze the dynamical transmission of Ebola virus is proposed. The basic reproduction number is defined, and the mathematical analysis on the local stability of the disease-free equilibrium and endemic equilibrium is given. By the least squares method and the recognized infectious and death cases in Guinea, parameters of the model are estimated and the estimated value of the basic reproduction number is obtained. The sensitivity and uncertainty property of the basic reproduction number is discussed by partial rank correlation coefficients.

## Figures and Tables

**Table 1:** The estimated values of model parameters by least square method (unit: year^−1^).

Parameters	Definition	Initial values	Estimates
β_10_	Contact rate in infectious period	5.0138 × 10^−11^	6.474 × 10^−11^
β_20_	Contact rate in latent and exposed period	9.3133 × 10^−8^	5.685 × 10^−9^
μ_20_	Disease-caused death rate in infectious period	0.5061	0.6647
ε_0_	Transfer rate from latent and exposed to the infectious	0.4	0.0596
*v*_0_	Transfer rate form the infectious to treatment state	0.5140	0.8613
γ_0_	Effective treated rate	0.2	0.2999

## Appendices

### A. Calculation of *b*_1_, *b*_2_, *b*_3_, *b*_4_

By (6), (16), and the definition of *A*, we obtain

(A.1) b1β1I∗+β2E∗−β2S∗+ω1+ω2+ω3+1=A−β2ω1ω2ɛβ1+ω2β2+ω1+ω2+ω3+1=A+ω1−ω1ω2β2ɛβ1+ω2β2+ω2+ω3+1=A+ɛω1β1ɛβ1+ω2β2+ω2+ω3+1.

Calculating *b*_2_, we have

(A.2) b2ω1+ω2+ω3A+1−1+ω2+ω3β2+ɛβ1S∗+ω1ω2+ω3+ω2ω3=ω1+ω2+ω3A+1−1+ω2+ω3β2+ɛβ1ω1ω2ɛβ1+ω2β2+ω1ω2+ω3+ω2ω3=ω1+ω2+ω3A+1+ɛω1ω3β1−ω1ω2β1ɛβ1+ω2β2+ω2ω3=ω1+ω2+ω3A+ω1ɛβ1+ω2β2+ɛω1ω3β1−ω1ω2β1ɛβ1+ω2β2+ω2+ω3+ω2ω3=ω1+ω2+ω3A+ɛω1β1+ɛω1ω3β1ɛβ1+ω2β2+ω2+ω3+ω2ω3.

For *b*_3_, we have

(A.3) b3ω2ω3+ω1ω2+ω3β1I∗+β2E∗−ω2ω3+ω2+ω3β2+ɛ1+ω3β1S∗+ω1ω2ω3+ω2+ω3+ω2ω3=ω2ω3+ω1ω2+ω3A+1−ω2ω3β2+ɛω3β1+ω2+ω3β2+ɛβ1S∗+ω1ω2ω3=ω2ω3+ω1ω2+ω3A+1−ω2+ω3β2+ɛβ1ω1ω2ɛβ1+ω2β2=ω2ω3+ω1ω2+ω3A+ω2ω3+ɛω1ω3β1ɛβ1+ω2β2.

Lastly, it is easy to get

(A.4) b4ω1ω2ω3−ɛγvβ1I∗+β2E∗=ω1ω2ω3−ɛγvA.

### B. Calculation of *b*_3_(*b*_1_ *b*_2_ − *b*_3_) − *b*_1_^2^ *b*_4_

By calculating, we have

(B.1) b3b1b2−b3=ω1+ω2+ω3ω2ω3+ω1ω2+ω3A3+ɛω1β1+ɛω1ω3β1ɛβ1+ω2β2ω2ω3+ω1ω2+ω3A2+ω2ω3+ω1ω2+ω3ω1+ω2+ω3A2+ɛω1β1ɛβ1+ω2β2ω2ω3+ω1ω2+ω3ω1+ω2+ω3A2+ω2+ω3·ω2ω3+ω1ω2+ω3ω2+ω3+1A2+ɛω1ω3β1ɛβ1+ω2β2ω1+ω2+ω3A2+ω1+ω2+ω3ω2ω3A2+ɛω1β1ɛβ1+ω2β2ω2+ω3+ω2ω3ω2ω3+ω1ω2+ω3A+ɛω1β1ɛω1β1+ɛω1ω3β1ɛβ1+ω2β22ω2ω3+ω1ω2+ω3A+ɛω1ω2β1+ɛω1ω2ω3β1ɛβ1+ω2β2ω2ω3+ω1ω2+ω3A+ɛω1ω32β1ɛβ1+ω2β2ω2ω3+ω1ω2+ω3A+ɛω1β1+ɛω1ω3β1ɛβ1+ω2β2ω2ω3+ω1ω2+ω3A+ω2+ω3ω2+ω3+1ω2ω3+ω1ω2+ω3A+ω2ω3ω2+ω3ω2ω3+ω1ω2+ω3A+ɛω1ω3β1ɛβ1+ω2β2ω1+ω2+ω3A+ɛω1β1+ɛω1ω3β1ɛβ1+ω2β2ω2ω3A+ω1+ω2+ω3·ω2ω3A+ɛω1β1ɛβ1+ω2β2ω1+ω2+ω3ω2ω3A+ω2+ω3ω2+ω3+1ω2ω3A+ɛω1ω3β1ɛβ1+ω2β2ω2+ω3ω2+ω3+1A+ɛω1ω3β1ɛω1β1+ɛω1ω3β1ɛβ1+ω2β22A+ε2ω12ω3β12ɛβ1+ω2β2ω1+ω2+ω3A+C1C2,

where

(B.2) C1=ω2ω2+ɛω1ω3β1ɛβ1+ω2β2,C2=ɛω1β1ɛβ1+ω2β2ω2+ω3+ω2ω3+ɛω1β1ɛω1β1+ɛω1ω3β1ɛβ1+ω2β22+ɛω1ω2β1+ɛω1ω2ω3β1ɛβ1+ω2β2+ɛω1ω32β1ɛβ1+ω2β2+ɛω1β1+ɛω1ω3β1ɛβ1+ω2β2+ω2+ω3ω2+ω3+1+ω2ω3ω2+ω3.

On the other hand,

(B.3) b12b4=ω1ω2ω3A3+2ɛω1β1ɛβ1+ω2β2ω1ω2ω3A2+2ω2+ω3+1ω1ω2ω3A2+ω2+ω3+12ω1ω2ω3A+ɛω1β1ɛβ1+ω2β22ω1ω2ω3A+2ɛω1β1ɛβ1+ω2β2ω2+ω3+1ω1ω2ω3A−ɛγvA3−2ɛω1β1ɛβ1+ω2β2ɛγvA2−2ω2+ω3+1ɛγvA2−ω2+ω3+12ɛγvA−ɛω1β1ɛβ1+ω2β22ɛγvA−2ɛω1β1ɛβ1+ω2β2ω2+ω3+1ɛγvA.

Therefore, we further have

(B.4) b3b1b2−b3−b12b4=ω1+ω2+ω3ω2ω3+ω1ω2+ω3A3−ω1ω2ω3A3+ɛω1β1ɛβ1+ω2β2ω2ω3+ω1ω2+ω3ω1+ω2+ω3A2−2ɛω1β1ɛβ1+ω2β2ω1ω2ω3A2+ω2+ω3·ω2ω3+ω1ω2+ω3ω2+ω3+1A2−2ω2+ω3+1ω1ω2ω3A2+ɛω1β1+ɛω1ω3β1ɛβ1+ω2β2ω2ω3+ω1ω2+ω3A2+ω2ω3+ω1ω2+ω3ω1+ω2+ω3A2+ɛω1ω3β1ɛβ1+ω2β2ω1+ω2+ω3A2+ω1+ω2+ω3·ω2ω3A2+ω2+ω3ω2+ω3+1ω2ω3+ω1ω2+ω3A+ω2ω3ω2+ω3ω2ω3+ω1ω2+ω3A−ω2+ω3+12ω1ω2ω3A+ɛω1β1ɛω1β1+ɛω1ω3β1ɛβ1+ω2β22ω2ω3+ω1ω2+ω3A−ɛω1β1ɛβ1+ω2β22ω1ω2ω3A+ɛω1β1ɛβ1+ω2β2ω2+ω3+ω2ω3ω2ω3+ω1ω2+ω3A+ɛω1ω2β1+ɛω1ω2ω3β1ɛβ1+ω2β2ω2ω3+ω1ω2+ω3·A−2ɛω1β1ɛβ1+ω2β2ω2+ω3+1ω1ω2ω3A+ɛω1ω32β1ɛβ1+ω2β2ω2ω3+ω1ω2+ω3A+ɛω1β1+ɛω1ω3β1ɛβ1+ω2β2ω2ω3+ω1ω2+ω3A+ɛω1ω3β1ɛβ1+ω2β2ω1+ω2+ω3A+ɛω1β1+ɛω1ω3β1ɛβ1+ω2β2ω2ω3A+ω1+ω2+ω3·ω2ω3A+ɛω1β1ɛβ1+ω2β2ω1+ω2+ω3ω2ω3A+ω2+ω3ω2+ω3+1ω2ω3A+ɛω1ω3β1ɛβ1+ω2β2ω2+ω3ω2+ω3+1A+ɛω1ω3β1ɛω1β1+ɛω1ω3β1ɛβ1+ω2β22A+ε2ω12ω3β12ɛβ1+ω2β2ω1+ω2+ω3A+ɛγvA3+2·ɛω1β1ɛβ1+ω2β2ɛγvA2+2ω2+ω3+1ɛγvA2+ω2+ω3+12ɛγvA+ɛω1β1ɛβ1+ω2β22ɛγvA+2ɛω1β1ɛβ1+ω2β2ω2+ω3+1ɛγvA+C1C2>ɛω1β1+ɛω1ω3β1ɛβ1+ω2β2ω2ω3+ω1ω2+ω3A2+ω2ω3+ω1ω2+ω3ω1+ω2+ω3A2+ɛω1ω3β1ɛβ1+ω2β2ω1+ω2+ω3A2+ω1+ω2+ω3·ω2ω3A2+ɛω1ω32β1ɛβ1+ω2β2ω2ω3+ω1ω2+ω3A+ɛω1β1+ɛω1ω3β1ɛβ1+ω2β2ω2ω3+ω1ω2+ω3A+ɛω1ω3β1ɛβ1+ω2β2ω1+ω2+ω3A+ɛω1β1+ɛω1ω3β1ɛβ1+ω2β2ω2ω3A+ω1+ω2+ω3·ω2ω3A+ɛω1β1ɛβ1+ω2β2ω1+ω2+ω3ω2ω3A+ω2+ω3ω2+ω3+1ω2ω3A+ɛω1ω3β1ɛβ1+ω2β2ω2+ω3ω2+ω3+1A+ɛω1ω3β1ɛω1β1+ɛω1ω3β1ɛβ1+ω2β22A+ε2ω12ω3β12ɛβ1+ω2β2ω1+ω2+ω3A+ɛγvA3+2·ɛω1β1ɛβ1+ω2β2ɛγvA2+2ω2+ω3+1ɛγvA2+ω2+ω3+12ɛγvA+ɛω1β1ɛβ1+ω2β22ɛγvA+2ɛω1β1ɛβ1+ω2β2ω2+ω3+1ɛγvA+C1C2>0.
