# Supplementary material for: Dynamical Analysis of an SEIT Epidemic Model with Application to Ebola Virus Transmission in Guinea
Source: Comput Math Methods Med. 2015 Jul 2;2015:582625. doi: 10.1155/2015/582625 (PMC4504125; doi:10.1155/2015/582625)
Supplement: Supplementary file 1 — This paper establishes an SEIT epidemic model (susceptible, exposed in the latent period, infectious, treated/recovery) to analyze the dynamical transmission of Ebola virus is proposed. The basic reproduction number is defined, and the mathematical analysis on the local stability of the disease-free equilibrium and endemic equilibrium is given. By the least squares method and the recognized infectious and death cases in Guinea, parameters of the model are estimated and the estimated value of the basic reproduction number is obtained. The sensitivity and uncertainty property of the basic reproduction number is discussed by partial rank correlation coefficients. [file 582625.f1.pdf]

Dynamical analysis of an SEIT epidemic model with application to Ebola virus transmission in Guinea

Highlights (for review)

- This paper introduces an SEIT epidemic model (susceptible, exposed in the latent period, infectious, treatment) to analyze the dynamical transmission of Ebola virus.
- The basic reproduction number is defined. The mathematical analysis on the existence and stability of the disease-free equilibrium and endemic equilibrium is given.
- By the least squares method and the recognized infectious and death cases in Guinea, parameters of the model are estimated and the estimated value of the basic reproduction number is obtained.
- The sensitivity and uncertainty property of the basic reproduction number is disscussed by partial rank correlation coefficients.
